# Multiplex PCR–Mass Spectrometry Mini-Sequencing Technology Detected Antibiotic Resistance of *Helicobacter pylori* to Six Antibiotics

**DOI:** 10.3390/ijms26041632

**Published:** 2025-02-14

**Authors:** Fei Zhao, Xin Zhao, Huifang Zhang, Lihua He, Fanliang Meng, Jianzhong Zhang, Di Xiao

**Affiliations:** National Key Laboratory of Intelligent Tracking and Forecasting for Infectious Diseases, National Institute for Communicable Disease Control and Prevention, Chinese Center for Disease Control and Prevention, Beijing 102206, China; zhaofei@icdc.cn (F.Z.); zhaoxin@icdc.cn (X.Z.); zhanghuifang@icdc.cn (H.Z.); helihua@icdc.cn (L.H.); mengfanliang@icdc.cn (F.M.); zhangjianzhong@icdc.cn (J.Z.)

**Keywords:** *Helicobacter pylori*, antibiotics, antibiotic resistance, mutation, multiplex PCR–mass spectrometry mini-sequencing technology

## Abstract

The abuse of antibiotics has led to widespread resistance to *Helicobacter pylori* (*H. pylori*) in the population. There is an urgent need to establish a method to detect multiple antibiotic resistance rapidly. This study aimed to construct a novel strategy for the high-throughput detection of *H. pylori*’s resistance to varying antibiotics using multiplex PCR–mass spectrometry mini-sequencing (mPCR-MS mini-sequencing) technology. This study detected the resistance of *H. pylori* to six antibiotics using eight mutated sites (23S *rRNA*-2143; *pbp1A*-1667, 1684, 1240; *gyrA*-261, 271, 573; and 16S *rRNA*-928) of four resistance genes (*pbp1A*, *gyrA*, 23S *rRNA*, and 16S *rRNA*), and 525 were detected in all 528 results (99.43%). Then, the culture-based phenotypic drug susceptibility testing (DST) method was used as a reference for drug resistance detection. We found that the consistency rate between mPCR-MS mini-sequencing with the DST results of amoxicillin (AMX), moxifloxacin (MOX), levofloxacin (LEV), clarithromycin (CLA), azithromycin (AZI), and tetracycline (TET) were 95.5% (63/66), 77.3% (51/66), 68.2% (45/66), 93.9% (62/66), 92.4% (61/66), and 97.0% (64/66), respectively. This method was high-throughput and extensible, easily improving the entire detection system by adding new mutation sites. mPCR-MS mini-sequencing technology provides a new approach to mutation sites related to *H. pylori*’s antibiotic resistance.

## 1. Introduction

*Helicobacter pylori* (*H. pylori*), a Gram-negative bacillus, infects about 50% of the world’s population, approximately 4.4 billion individuals [1]. *H. pylori* infection often does not cause any symptoms, and about 1–10% of infected patients develop clinical symptoms, including peptic ulcer disease, gastric atrophy, gastric intestinal metaplasia, and ultimately, gastric cancer or mucosa-associated lymphoid tissue (MALT) lymphoma [2]. *H. pylori* is a potent carcinogen, and the eradication of *H. pylori* infection has shown positive effects in decreasing the risk of gastric cancer.

There are certain difficulties in eradicating *H. pylori* associated with its treatment, and a single antibiotic cannot achieve a therapeutic effect. The approach for treating *H. pylori* infection diverges considerably from treatment protocols used for other infectious diseases. Eliminating *H. pylori* necessitates a meticulous blend of multiple medications, primarily antibiotics and acid inhibitors, as the cornerstone regimen. The choice between quadruple and triple therapy as initial treatment hinges critically on the clarithromycin resistance rate exceeding 15% [3,4]. In cases where the initial treatment proves unsuccessful, alternative therapeutic avenues are evaluated [5].

Regrettably, the effectiveness of empirical therapy has been declining, primarily owing to the escalating antimicrobial resistance and the shifting epidemiological landscape. Notably, antimicrobial resistance has emerged as one of the foremost contributors to treatment ineffectiveness [6]. Specifically, the treatment of *H. pylori* should consider local and individual antibiotic resistance patterns. Moreover, it is essential to conduct antimicrobial resistance surveillance through bacterial culture or molecular methods to determine the most effective therapy for managing *H. pylori* infection [7]. Due to the difficulty and inconsistent success in isolating *H. pylori* from gastric biopsy samples, bacterial culture for antibiotic resistance is not always feasible [8].

Combination therapy for *H. pylori* includes proton pump inhibitors (PPIs) and gastric mucosal protectors with one or two antibiotics to form triple or quadruple therapy. Moreover, a few antibiotics, such as amoxicillin (AMX), moxifloxacin (MOX), levofloxacin (LEV), clarithromycin (CLA), azithromycin (AZI), and tetracycline (TET), and metronidazole (MET), effectively eradicate *H. pylori* [7]. These antibiotics are widely used in the population, increasing antibiotic resistance yearly [2,9]. Therefore, it is necessary to detect antibiotic resistance rapidly, which is conducive to precision medication to improve the eradication rate.

The reference method for detecting *H. pylori* resistance is still solid culture-based drug susceptibility testing (DST). DST has limitations because *H. pylori* is a slow-growing microaerobic bacteria, and which requires a relatively long time to identify multiple antibiotics [10]. The DST method includes agar dilution, gradient strip diffusion, disk diffusion, and broth microdilution testing. Agar dilution as the reference method for *H. pylori* susceptibility testing is time-consuming, labor-intensive, and expensive. Other DST methods have many limitations; the inoculum density, medium used, antibiotic disk content, incubation conditions, and zone diameter measurement criteria may contribute to variability [11]. Several molecular assays provide the possibility of rapid detection of antibiotic resistance. Whole genome sequencing (WGS) provides more comprehensive drug resistance information, but it is expensive and difficult to perform in many laboratories due to a lack of resources and expertise. Polymerase chain reaction (PCR) and quantitative PCR (qPCR) have poor coverage of different antibiotics and are mostly used to detect a few key antibiotic resistance genes. Molecular assays currently used in clinical practice include CLA and LEV [12,13,14]. Multisite detection methods of antibiotic resistance are urgently needed.

In recent decades, studies have demonstrated that a multitude of drug resistance mechanisms are associated with the emergence of antibiotic resistance in *H. pylori*, with genetic mutations playing a prominent role among them [6,15,16]. Matrix-assisted laser desorption ionization–time of flight mass spectrometry (MALDI-TOF MS) has shown great potential in nucleic acid detection and analysis, and it has been used in the study of single nucleotide polymorphisms (SNPs), gene mutations, deoxyribonucleic acid (DNA) methylation, and DNA copy number variation. The basic process is as follows: amplifying the template DNA through multiplex PCR and performing single-base extension after shrimp alkaline phosphatase (SAP) treatment. MALDI-TOF MS is performed to identify the increase in mass-to-charge ratio (*m*/*z*) after single-base extension. To understand and distinguish it from other mass spectrometry techniques, our group named this technique of detecting SNPs by multiplex PCR coupled with MALDI-TOF MS ‘multiplex PCR–mass spectrometry mini-sequencing (mPCR-MS mini-sequencing) technology’ [17]. This detection method has been widely used in typing and identifying pathogenic microorganisms, drug resistance detection, and drug susceptibility [17,18,19].

In this study, using mPCR-MS mini-sequencing technology, a sixfold PCR system was constructed to detect the resistance to six antibiotics used to eradicate *H. pylori*. The reliability of the detection method was evaluated in 66 strains.

## 2. Results

### 2.1. Establishment and Optimization of mPCR-MS Mini-Sequencing Technology

**Figure 1:**
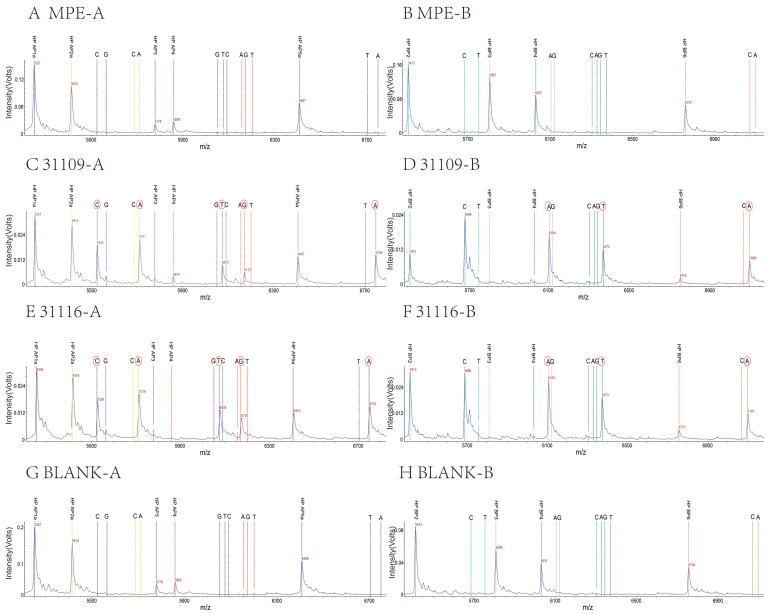
MS peaks of the MPE probes in different samples. (**A**,**B**): MS peaks of MPE probes without extension; (**C**,**D**): SNP peaks of MPE probes extended in strain 31109; (**E**,**F**): SNP peaks of MPE probes extended in strain 31116; (**G**,**H**): MS peaks of MPE probes without extension in blank control. Colors: each color represents one MPE and its extension bases. Letters: the letters denote the types of bases the MPE probes can extend. Red circles: the circles represent the bases extended by the MPE probes in the strains.

The eight target sites were amplified using six-fold PCR. The m/z of the mass probe extension (MPE) original peaks for eight mutation types (HP AP1a/b, HP AP2a/b, HP AP3, HP AP4, HP AP5a/b, HP BP3, HP BP4, and HP BP6) were 5251.4 ± 3, 5413.6 ± 3, 5777.8 ± 3, 5858.8 ± 3, 6407.2 ± 3, 5807.8 ± 3, 6031.0 ± 3, and 6756.4 ± 3 (Table 1), respectively (mass error less than 500 ppm, the same below). We established the method using the WGS strains numbered ICDC 31109 and ICDC 31116; the A-tube extension results of 31109 and 31116 strains were detected by mass spectrometry at 5524.4 ± 3, 5710.6 ± 3, 6074.8 ± 3, 6171.8 ± 3, and 6749.2 ± 3, and the extension bases were C, A, T, G, and A. The B-tube extension results of the 31109 and 31116 strains were 6104.8, 6373.0, and 7098.4, and the extension bases were A, T, and A. The results were consistent with the WGS results. There were no MS peaks in the blank tube, which was used to replace the template DNA with DNase-free water during PCR amplification. The mass spectrometry result also showed non-extension in the negative control tube, replacing the SAP product with DNase-free water during the MPE reaction (Table 1 and Table 2, Figure 1).

After optimization, the final concentrations of the eight MPEs were 7.14 μM (HP AP1a/b), 7.44 μM (HP AP2a/b), 8.06 μM (HP AP3), 8.19 μM (HP AP4), 9.05 μM (HP AP5a/b), 8.11 μM (HP BP3), 8.47 μM (HP BP4), and 9.56 μM (HP BP6).

### 2.2. Antibiotic Susceptibility Testing of H. pylori Strains

**Figure 2:**
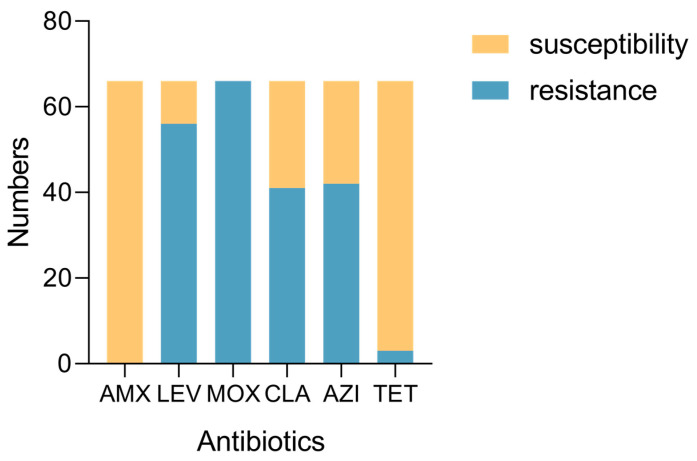
The numbers of resistance or susceptibility to six antibiotics.

We assessed the antimicrobial susceptibility of 66 *H. pylori* strains to six antibiotics, AMX, LEV, MOX, CLA, AZI, and TET. The clinical breakpoints of each antibiotic were as follows: AMX, 1 mg/L, LEV, 1 mg/L, MOX, 1 mg/L, CLA, 1 mg/L, AZI, 1 mg/L, and TET, 4 mg/L. We judged whether resistance (R) and susceptibility (S) were based on the clinical breakpoints of each antibiotic. We found that 0% of all strains were resistant to AMX (0/66), 84.8% were resistant to LEV (56/66), 100% were resistant to MOX (66/66), 62.1% were resistant to CLA (41/66), 63.6% were resistant to AZI (42/66), and 0.45% were resistant to TET (3/66) (Figure 2, Table 3).

### 2.3. Eight Mutation Sites of Sixty-Six Strains Were Detected by mPCR-MS Mini-Sequencing Technology

**Figure 3:**
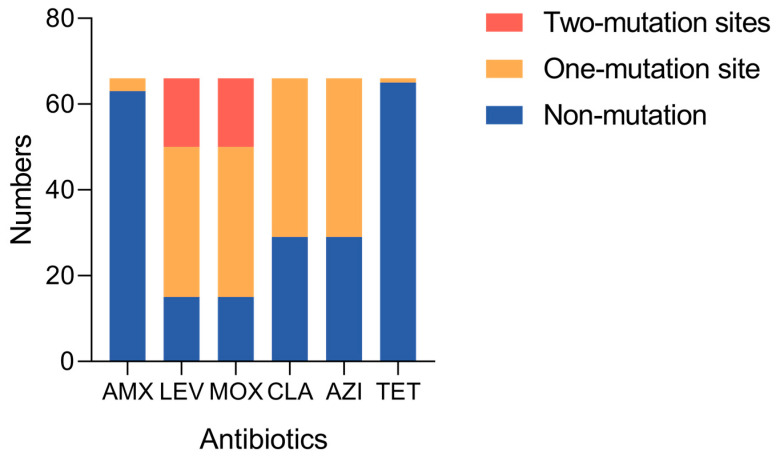
The number of mutations at mutation sites associated with six antibiotics.

Eight mutation sites were detected among the 66 *H. pylori* strains, and 525 were detected in all 528 mutation sites (99.43%). The detection rate of three mutation sites—the penicillin-binding proteins (*pbp1A*)-A1240C, C1667G, and A1684T of AMX—was 95.5% (63/66). The mutation rate of A1684T of *pbp1A* was 4.5% (3/66). There were three mutation sites—DNA gyrase subunit A (*gyrA*)-C261TAG, G271AT, and T573GC of LEV and MOX—with a 100% (66/66) detection rate. There was a 77.3% (51/66) mutation rate of *gyrA*. The most common mutation was C261TAG, present in 75.8% (50/66), the second-most common was G271AT, present in 24.2% (16/66), and the last one was T573GC, present in 1.5% (1/66). One mutation site, 23S ribosomal ribonucleic acid (*rRNA*)-A2143G, was tested for CLA and AZI, which had a 100% (66/66) detection rate and a 56.1% (37/66) mutation rate of 23S *rRNA*. A928C of 16S *rRNA* had a 100% (66/66) detection rate for testing the resistance of TET; only one mutation was detected in the 66 strains (1.5%) (Figure 3, Table 4).

The mass spectrometry test results showed bimodal peaks at four sites of eight strains (Table 4). These sites were compared using PCR combined with Sanger sequencing, and the sequencing results were bimodal: site 271 in strains 11050, 11091, and 12104; site 261 in strains 11091, 11085, 12057, 22090, 12104, and 31123; site 1684 in strain 11039; and site 2143 in strain 11050 (Table 4, Figure S1).

### 2.4. Consistency Rate of DST and mPCR-MS Mini-Sequencing Technology

DST methods were the reference for drug resistance detection in this study. Our paper detected the resistance of 66 strains to six antibiotics through DST and mass spectrometry detection of mutations. The consistency rate is [(true positive rate + true negative rate)/total number of tests × 100%]. These results suggest that the consistency rates of mass spectrometry detection of mutations with MICs of AMX, MOX, LEV, CLA, AZI, and TET were 95.5% (63/66), 77.3% (51/66), 68.2% (45/66), 93.9% (62/66), 92.4% (61/66), 97.0% (64/66), respectively (Table 5).

## 3. Discussion

In this endeavor, we devised mPCR-MS mini-sequencing technology that concurrently identifies resistance loci for six crucial drugs (AMX, MOX, LEV, CLA, AZI, and TET), and we judged drug resistance on the basis of mutational signatures within patient samples. Our method underwent rigorous validation, comparing the mutational profiles of these resistance sites with DST outcomes; notably, for AMX, MOX, LEV, CLA, AZI, and TET, the concordance between this molecular approach and DST result ranged from 68.2% to 97.0%. These results underscore the reliability and accuracy of our innovative detection strategy. The consistency rate of these two methods of AMX, CLA, AZI, and TET drug resistance detection was higher (95.5%, 93.9%, 92.4%, and 97.0% respectively). Among them, mPCR-MS mini-sequencing technology detected fewer strains containing AMX and TET resistance-related mutations (3/66 and 1/66, respectively), which was more in line with the low AMX and TET drug resistance rates in the clinical context [8]. At the same time, mPCR-MS mini-sequencing technology detected the presence of CLA and AZI resistance-related mutations (37/66) in multiple strains, which was more in line with the results of the current high clinical CLA drug resistance rate [8]. MPCR-MS mini-sequencing technology provides a novel strategy to detect resistance in patients to AMX, CLA, AZI, and TET. The detection method we constructed had a lower consistency rate regarding the DST results of MOX and LEV (77.3% and 68.2%, respectively), and the system needs to be further optimized.

In this study, no drug resistance was detected to the MIC of AMX by DST, but three isolates were detected by mPCR-MS mini-sequencing technology with mutations. Among them, the 11039 strain was identified with site 1684 as AT, and the other two strains, 12104 and 11022, were detected as T. According to the Sanger sequencing and mPCR-MS mini-sequencing results, it was determined to be an AMX-mutant strain, but these MIC results were sensitive. These three sites—1240, 1667, and 1684—represented the amino acid sites 414, 556, and 562 of *pbp1A*. According to previous research, several amino acid variations in or adjacent to the second and third conserved penicillin-binding protein motifs (PBM) could mediate AMX resistance in *H. pylori* [20,21]. Hence, 414 (site 1240, adjacent to the second PBM) and 556 (site 1667, located in the third PBM) mutations represented the major factors in resistance. Our results indicated that the three strains, which possessed only site 1684 mutations, were insufficient to induce amoxicillin resistance. In addition, it was postulated that potential synergistic interactions among other drug-resistant genes could facilitate the manifestation of drug resistance. Mutations in *pbp2*, *pbp3*, *hefC*, *hopC*, and *lofH* were also related to the resistance of *H. pylori* to AMX, and *pbp1A* may have a synergistic effect with these genes [2,22,23].

MOX and LEV are quinolone antibiotics, and the resistance rate of *H. pylori* to quinolone antibiotics in China is extremely high [24]. The consistency rate between mPCR-MS mini-sequencing and MICs of LEV was 68.2% (45/66). Eight strains with no phenotypic resistance were observed in the presence of *gyrA* sequence mutations. These results could be attributed to the LEV resistance breakpoint utilized in this study (1 μg/mL). Six of the eight sensitivity judgments had MIC values of 0.75 μg/mL, close to the drug resistance limit of 1 μg/mL. Other research reported isolates harboring *gyrA* mutations with a MIC of 0.5 μg/mL [25]. Additional studies are needed to correlate MIC and *gyrA* mutations to obtain an accurate breakpoint for levofloxacin [26]. No mutations were identified in 13 strains with phenotypic resistance in this study. The detected mutation sites C261TAG, G271AT, and T573GC in the *gyrA* gene correspond to the following *gyrA* amino acid sites: N87/N87K, D91/D91N/D91Y, and I191/I191M. Other studies have evaluated *H. pylori* genotypic resistance to LEV, where N87I, N87K, D91N, D91Y, and D91G were major mutations [26,27,28]. In this study, no MPE was designed for mutation N87I and D91G in our samples. We found in the pre-experiment that adding these two mutant probes affected the system stability. Previous retrospective studies have shown that the proportion of N87K in China is higher [29,30,31]. Moreover, mutations in *gyrB*, such as N481E and R484K, were also related to the resistance of *H. pylori* to LEV [32].

23S *rRNA* sequencing was performed on five inconsistent strains (12073, 12132, 21104, 22098, and 11039), of which the sequencing results of 12073 and 12132 showed that both were A2142G mutations. In the detection system employed for this study, we encountered nonspecific outcomes in identifying A2142G. Given the notably low detection rate of A2142G, which fell below 5%, the site was subsequently excluded from our analysis, with the focus solely directed toward detecting A2143G [33]. The remaining three strains (21104, 22098, and 11039) exhibited divergent DST outcomes when evaluated against CLA and AZI. However, mPCR-MS mini-sequencing technology indicated no mutations at sites 2142 and 2143. This observation suggests that the resistance might be attributed to mutations occurring at different sites. For instance, mutations such as C2694A and T2717C, which have been associated with lower resistance levels, may be contributing factors. Consequently, these particular sites may not serve as definitive drug resistance determinants [34,35].

The results detected by mPCR-MS mini-sequencing technology showed that isolates 12086 and 11039 were wild type A. The MIC result of 11039 to TET was the critical value, probably a human misjudgment. While the result of 12086 may be related to mutations in other missed detection locations, AGA926-928TTC was the major mutation of TET [36,37]. Combining large doses of TET and AMX could effectively eradicate *H. pylori*, and the incidence of adverse events was lower than that for programs containing furazolidone [38]. However, due to the low resistance of *H. pylori* to TET, the results of this experiment also supported this view, so it was recommended as a first-line drug for *H. pylori* infection in many countries, including China and the United States [4,39].

MET is the first-line therapy for *H. pylori* [4]. Recently, the results of some studies have indicated that the primary resistance rate to MET is 40–70% in China [24]. The mechanism of MET resistance is very complex [40,41,42]. It involves a wide range of genes and mutant forms, and many sites are still controversial [37,43]. In this study, only one relative mutation, C148T of *rdxA*, was selected for our experiment, and the results showed that the three isolates (strains 31073, 22103, and 22075) mutated at this site were both drug-resistant strains (Table S1). The primers, MPE probe, and extension bases are provided in the supplementary materials (Table S2).

We detected bimodal peaks at four sites of eight strains. The sites (site 271 of strains 11050, 11091, and 12104; site 261 of strains 11091, 11085, 12057, 22090, 12104, and 31123) of the *gyrA* associated with MOX and LEV resistance were detected as bimodal, and the DST results of these strains showed drug resistance (MIC > 32 mg/L). Site 2143 of 23S *rRNA* associated with CLA and AZI resistance was detected as bimodal, and the DST results of the 11050 strain showed drug resistance (MIC > 32 mg/L) too. The detected site 1684 of *pbp1A* associated with AMX was bimodal, and the DST results of these strains showed drug susceptibility (MIC < 1). Our results showed that when mPCR-MS mini-sequencing technology detected bimodal calls (one wild type, one mutant type, and two mutant types), there was a high probability of antibiotic resistance, indicating that they should be avoided. AMX with low drug resistance may be considered for use.

Compared with previous research, our research not only included the CLA and LEV drug resistance genes 23S *rRNA* and *gyrA* but also four other antibiotic-related drug resistance genes. What was inconsistent with some studies was the A2143G in the 23S *rRNA* selected in this study, not A2142G/C [14]. This study overlooked certain mutations associated with antibiotic resistance, namely *gyrA*-N87I and 23S *rRNA*-A2142GC et. The detection system should be optimized to encompass a broader range of mutations linked to antibiotic resistance. Furthermore, this approach was confined to detecting predetermined mutations and SNPs exclusively in pure samples. MPCR-MS mini-sequencing technology has certain limitations for detecting drug resistance in mixed samples. It detected the presence of a mutation in a specific drug-resistance gene in the mixed samples. It was not possible to determine which sample it was. It could be used for regional antibiotic resistance screening. If there is a specific mutation related to antibiotic resistance in several mixed samples, it may indicate that the local resistance rate of this kind of antibiotic is high. However, there are individual differences in antibiotic resistance, and screening of mixed samples is not recommended in clinical detection.

Despite these limitations, the mPCR-MS mini-sequencing technology is a valuable bridge between PCR-coupled Sanger and WGS. Its strengths lie in its swift analysis, heightened sensitivity, moderate throughput, and minimal sample consumption (96 samples with less than 50 sites could be detected within 7 h) [17]. With future refinements to the system and advancements in instrumentation, it holds immense potential to serve as a benchmark for clinical laboratories, facilitate rapid assessment of *H. pylori* drug resistance, and guide personalized medication strategies tailored to individual resistance profiles.

## 4. Materials and Methods

### 4.1. H. pylori Strains

Two WGS strains, ICDC 31109 and ICDC 31116, and 66 *H. pylori* strains were obtained from the Institute of National Institute for Communicable Disease Control and Prevention, Chinese Center for Disease Control and Prevention (ICDC).

### 4.2. Antibiotic Susceptibility Testing

We determined the MIC using the Etest (Liofilchem, Teramo, Italy) to measure the susceptibility of the 66 *H. pylori* strains to six antibiotics, including AMX, LEV, MOX, CLA, AZI, and TET. The isolates were subcultured three times from −80 °C bacterial stock in a blood agar plate without antibiotics in microaerophilic conditions. On day two, *H. pylori* cultures were collected in normal saline buffer and then adjusted to a turbidity of 3.0 McFarland units using a McFarland turbidity meter. The *H. pylori* cultures were inoculated into Mueller Hinton agar (Becton Dickinson, Isère, France) supplemented with 5% sheep blood, and the E-test strip was placed in the middle of the plate. The evaluation was performed after 72 h of incubation in microaerophilic conditions (5–10% CO_2_, 5% O_2_, 85% N_2_, and 5% H_2_ generated by gas-generating sachets at 37 °C) [17]. The clinical breakpoint of each antibiotic was as follows: AMX, 1 mg/L, LEV, 1 mg/L, MOX, 1 mg/L, CLA, 1 mg/L, AZI, 1 mg/L, TET, 4 mg/L.

### 4.3. H. pylori DNA Preparation

*H. pylori* DNA was isolated from *H. pylori* clinical isolation using QIAamp^®^ DNA Micro (Qiagen, Dusseldorf, The Netherlands). The bacteria cultured overnight in 1 mL were centrifuged at 12,000 rpm for 1 min, and the supernatant was discarded. The steps were completed according to the kit’s protocol: the centrifuge column bound the bacterial DNA, washed it to remove impurities, eluted it from the centrifuge column, collected it, and stored it at −20 °C.

### 4.4. Selected Target Genes and Designed Primers and Mass Probes for Antibiotic Resistance

The design principles of multiplex-PCR primers have been previously described [17,18]. Initially, NCBI software (version hg38) retrieved resistance genes (*pbp1A*, *gyrA*, 23S *rRNA*, and 16S *rRNA*) sequences of standard strains and other thirty-odd uploaded strains (Table 6). A tool named Alignment was selected for sequence alignment. Eight mutation sites were chosen for antibiotic resistance, namely 23S *rRNA*-2143; *pbp1A*-1667, 1684, and 1240; *gyrA*-261, 271, and 573; and 16S *rRNA*-928 (Table 7). The conserved segment was analyzed using BatchPrimer3 (version 1.0) software (https://wheat.pw.usda.gov/demos/BatchPrimer3/, accessed on 18 November 2023) to design primers for multiplex PCR. Six primers were used for multiplex PCR, in which a 10 bp fixed sequence (acgttggatg) was added to the 5′ end of each primer (Table 8). Eleven quality difference probes for mutation sites were devoted into two groups, The probes HP AP1a, HP AP1b, HP AP2a, HP AP2b, HP AP3, HP AP4, HP AP5a, and HP AP5b were extended in one reaction tube (denoted as tube A), and probes HP BP3, HP BP4, and HP BP6 were extended in another reaction tube (denoted as tube B)→(Table 7).

### 4.5. Establishment of mPCR-MS Mini-Sequencing Technology

DNase-free water served as the blank control. The steps were as follows. (i) For multiplex PCR amplification, the mutated gene fragment was amplified. (ii) For SAP digestion, PCR products were treated with SAP to eliminate the free deoxynucleotide triphosphates. (iii) For the MPE reaction, the purified SAP products were added with mixed MPE reaction for the single base extension. The specific steps were previously described [18].

### 4.6. SNP Identification and Data Analysis by MALDI-TOF MS

The products from the MPE reaction were purified using the ion-exchange resin. An aliquot (0.2–1 µL) of purified products was spotted onto a matrix preliminarily dried on the MALDI target. The matrix employed was a saturated solution of 3-hydroxypicolinic acid (3-HPA). All solvents were of a quality suitable for mass spectrometry. The parameters and data analysis have been described previously [17,18,19].

### 4.7. Statistical Analysis

To calculate the consistency rate of detection results between the mPCR-MS mini-sequencing technology and the reference method DST, the calculation method of the consistency rate was [(true positive rate + true negative rate)/total number of tests × 100%].

## Figures and Tables

**Table 1:** Sequences and extension calls of mass probe.

Mass Probes	Mutations	Mass Probe Sequences (5′-3′)	Mass Probe Mass (Da)	Extension Call	Mass Probe Mass (Da)	Extension Call	Mass Probe Mass (Da)	Extension Call	Mass Probe Mass (Da)	Extension Call	Mass Probe Mass (Da)
HP AP1a	C/G	AGAAATTGCCGGTAAAA	5251.4	C	5524.4	G	5564.4				
HP AP1b	C/G	AGAAATCGCTGGTAAAA	5251.4	C	5524.4	G	5564.4				
HP AP2a	A/C	AACCACGCATGGCACCCT	5413.6	A	5710.6	C	5686.6				
HP AP2b	A/C	AATCACGCATGGCACCCC	5413.6	A	5710.6	C	5686.6				
HP AP3	T/G/C	AAGCGTCTATGATTTCATC	5777.8	T	6074.8	G	6050.8	C	6090.8		
HP AP4	G/A/T	TGGCGATAACGCGGTTTAT	5858.8	G	6171.8	A	6155.8	T	6200.8		
HP AP5a	A/T	CAATGAACCAAGCGTCAATAT	6407.2	A	6749.2	T	6704.2				
HP AP5b	A/T	CAATGAACCAGGCATCAATAT	6407.2	A	6749.2	T	6704.2				
HP BP3	A/G	CTACCCGCGGCAAGACGGA	5807.8	A	6104.8	G	6120.8				
HP BP4	C/A/T/G	TACCACCCCCATGGCGATAA	6031.0	C	6304.0	A	6328.0	T	6373.0	G	6344.0
HP BP6	A/C	CCTAGGTAAGGTTCTTCGTGTA	6756.4	A	7098.4	C	7069.4				

**Table 2:** Results of two WGS strains (ICDC 31109 and ICDC 3111) detected by mPCR-MS mini-sequencing technology.

Sample Number	Information	Code1	Result1	Code2	Result2	Code3	Result3	Code4	Result4	Code5	Result5
A-31109	HP-MPE-A	HP AP1a	C	HP AP2a	A	HP AP3	T	HP AP4	G	HP AP5a	A
A-31116	HP-MPE-A	HP AP1a	C	HP AP2a	A	HP AP3	T	HP AP4	G	HP AP5a	A
MPE-A(NTC)	HP-MPE-A	HP AP1a	NoCall	HP AP2a	NoCall	HP AP3	NoCall	HP AP4	NoCall	HP AP5a	NoCall
BLANK-A	HP-MPE-A	HP AP1a	NoCall	HP AP2a	NoCall	HP AP3	NoCall	HP AP4	NoCall	HP AP5a	NoCall
B-31109	HP-MPE-B	HP BP3	A	HP BP4	T	HP BP6	A				
B-31116	HP-MPE-B	HP BP3	A	HP BP4	T	HP BP6	A				
MPE-B(NTC)	HP-MPE-B	HP BP3	NoCall	HP BP4	NoCall	HP BP6	NoCall				
BLANK-B	HP-MPE-B	HP BP3	NoCall	HP BP4	NoCall	HP BP6	NoCall				

**Table 3:** The DST results for six antibiotics tested on sixty-six strains.

Strains	DST Results											
	AMX (≥1 mg/L)	R/S	LEV (≥1 mg/L)	R/S	MOX (≥1 mg/L)	R/S	CLA (≥1 mg/L)	R/S	AZI (≥1 mg/L)	R/S	TET (≥4 mg/L)	R/S
32141	0.032	S	>32	R	>32	R	0.19	S	0.19	S	2	S
32130	<0.016	S	2	R	1.5	R	0.19	S	3	R	0.38	S
32126	<0.016	S	1.5	R	1.5	R	0.75	S	0.125	S	0.38	S
32115	<0.016	S	>32	R	>32	R	12	R	>256	R	0.19	S
32078	<0.016	S	0.5	S	3	R	0.064	S	0.19	S	0.38	S
32041	0.023	S	>32	R	>32	R	4	R	>256	R	0.75	S
31135	0.016	S	2	R	3	R	0.125	S	0.75	S	0.75	S
31123	<0.016	S	>32	R	>32	R	24	R	>256	R	0.38	S
31078	0.064	S	>32	R	>32	R	0.19	S	0.19	S	1	S
31073	0.016	S	>32	R	>32	R	0.125	S	0.125	S	0.5	S
31058	0.023	S	>32	R	>32	R	6	R	>256	R	0.25	S
22105	<0.016	S	0.75	S	2	R	0.125	S	0.125	S	0.38	S
22104	0.094	S	>32	R	>32	R	1.5	R	0.75	S	1	S
22103	0.016	S	>32	R	>32	R	>256	R	>256	R	0.38	S
22100	<0.016	S	12	R	>32	R	64	R	>256	R	0.25	S
22098	0.016	S	0.75	S	3	R	1	R	2	R	0.5	S
22096	<0.016	S	>32	R	>32	R	0.25	S	0.125	S	0.25	S
22092	0.125	S	>32	R	>32	R	16	R	>256	R	0.25	S
22090	<0.016	S	>32	R	>32	R	4	R	>256	R	1.5	S
22089	<0.016	S	6	R	>32	R	16	R	>256	R	1	S
22075	<0.016	S	>32	R	>32	R	4	R	>256	R	0.38	S
22070	<0.016	S	>32	R	>32	R	0.047	S	0.094	S	0.25	S
22061	<0.016	S	>32	R	>32	R	48	R	>256	R	1.5	S
22059	0.047	S	>32	R	>32	R	12	R	>256	R	6	R
22058	<0.016	S	1.5	R	1.5	R	12	R	>256	R	0.125	S
22051	0.094	S	>32	R	>32	R	16	R	>256	R	1	S
22049	<0.016	S	>32	R	>32	R	48	R	>256	R	0.38	S
22027	<0.016	S	0.38	S	>32	R	>256	R	>256	R	0.094	S
22024	<0.016	S	0.75	S	3	R	0.19	S	0.064	S	0.19	S
21076	0.023	S	32	R	>32	R	24	R	>256	R	1	S
21072	0.064	S	6	R	>32	R	12	R	>256	R	0.5	S
21062	0.016	S	0.75	S	2	R	0.19	S	0.19	S	1	S
21018	<0.016	S	>32	R	>32	R	8	R	>256	R	0.38	S
12142	<0.016	S	1	R	1.5	R	0.25	S	0.19	S	0.25	S
12137	<0.016	S	1	R	1.5	R	8	R	>256	R	0.5	S
12133	0.023	S	1	R	2	R	0.064	S	0.094	S	0.38	S
12132	<0.016	S	0.75	S	8	R	24	R	>256	R	0.75	S
12130	<0.016	S	0.75	S	1	R	0.125	S	0.19	S	0.5	S
12128	<0.016	S	>32	R	>32	R	>256	R	>256	R	1.5	S
12122	<0.016	S	0.75	S	3	R	0.047	S	0.094	S	0.5	S
12118	<0.016	S	>32	R	>32	R	0.75	S	0.75	S	0.19	S
12114	<0.016	S	>32	R	>32	R	16	R	>256	R	0.25	S
12110	0.064	S	>32	R	>32	R	0.125	S	0.5	S	0.25	S
12104	0.047	S	>32	R	>32	R	>256	R	>256	R	0.75	S
12094	<0.016	S	>32	R	>32	R	48	R	>256	R	1	S
12086	0.38	S	>32	R	>32	R	12	R	>256	R	12	R
12073	<0.016	S	0.25	S	1.5	R	96	R	>256	R	0.125	S
12058	0.047	S	1	R	4	R	0.19	S	0.094	S	0.5	S
12057	0.032	S	>32	R	>32	R	0.19	S	0.19	S	0.5	S
12050	0.047	S	1	R	1.5	R	0.047	S	0.38	S	0.5	S
12048	<0.016	S	>32	R	>32	R	6	R	>256	R	0.75	S
11142	0.023	S	>32	R	>32	R	24	R	>256	R	0.75	S
11091	<0.016	S	>32	R	>32	R	>256	R	>256	R	0.5	S
11085	<0.016	S	>32	R	>32	R	0.19	S	0.19	S	0.75	S
11083	<0.016	S	2	R	3	R	16	R	>256	R	0.25	S
11077	0.047	S	3	R	4	R	32	R	>256	R	0.5	S
11073	0.016	S	>32	R	>32	R	0.094	S	0.38	S	0.5	S
11072	<0.016	S	>32	R	>32	R	0.38	S	0.19	S	1	S
11071	0.064	S	>32	R	>32	R	>256	R	>256	R	1	S
11065	<0.016	S	>32	R	>32	R	16	R	>256	R	0.25	S
11057	<0.016	S	>32	R	>32	R	12	R	>256	R	2	S
11056	<0.016	S	>32	R	>32	R	>256	R	>256	R	1	S
11050	<0.016	S	>32	R	>32	R	32	R	>256	R	0.38	S
11039	0.25	S	>32	R	>32	R	0.75	S	8	R	4	R
11022	0.047	S	6	R	>32	R	16	R	>256	R	0.38	S
2402	0.023	S	>32	R	32	R	48	R	>256	R	2	S

**Table 4:** The results of eight mutation sites in sixty-six strains detected by mPCR-MS mini-sequencing technology.

Strains	Mutation Sites							
		AMX			LEV/MOX		CLA/AZI	TET
	*pbp1A*	*pbp1A*	*pbp1A*	*gyrA*	*gyrA*	*gyrA*	23S *rRNA*	16S *rRNA*
	A1240C	C1667G	A1684T	C261TAG	G271AT	T573GC	A2143G	A928C
32141	A	C	A	A	G	T	A	A
32130	A	C	A	T	G	T	A	A
32126	A	C	A	T	G	T	A	A
32115	A	C	A	C	G	T	G	A
32078	A	C	A	T	G	T	A	A
32041	A	C	A	T	G	T	G	A
31135	A	C	A	C	G	T	A	A
31123	A	C	A	T/G	G	T	G	A
31078	A	C	A	T	G	T	A	A
31073	A	C	A	T	A	T	A	A
31058	A	C	A	C	G	T	G	A
22105	A	C	A	T	G	T	A	A
22104	A	C	A	C	A	T	A	A
22103	A	C	A	C	G	T	G	A
22100	A	C	A	T	G	T	G	A
22098	A	C	A	T	G	T	A	A
22096	A	C	A	C	G	T	A	A
22092	A	C	A	G	G	T	G	A
22090	A	C	A	A/C	G	T	G	A
22089	A	C	A	C	G	T	G	A
22075	A	C	A	A	G	T	G	A
22070	A	C	A	T	A	T	A	A
22061	A	C	A	A	G	T	G	A
22059	A	C	A	C	G	T	G	C
22058	A	C	NoCall	C	G	T	G	A
22051	A	C	A	A	G	T	G	A
22049	A	C	A	A	G	T	G	A
22027	A	C	A	T	G	T	G	A
22024	A	C	A	C	G	T	A	A
21076	A	C	A	A	G	G	G	A
21072	A	C	A	T	G	T	G	A
21062	A	C	A	T	G	T	A	A
21018	A	C	A	T	G	T	G	A
12142	A	C	A	C	G	T	A	A
12137	A	NoCall	A	T	T	T	G	A
12133	A	C	A	T	G	T	A	A
12132	A	C	A	A	G	T	A	A
12130	A	NoCall	A	T	T	T	A	A
12128	A	C	A	G	G	T	G	A
12122	A	C	A	C	G	T	A	A
12118	A	C	A	A	G	T	A	A
12114	A	C	A	C	G	T	G	A
12110	A	C	A	T	G	T	A	A
12104	A	C	T	T/C	G/A	T	G	A
12094	A	C	A	G	G	T	G	A
12086	A	C	A	C	G	T	G	A
12073	A	C	A	T	G	T	A	A
12058	A	C	A	T	G	T	A	A
12057	A	C	A	A/C	G	T	A	A
12050	A	C	A	C	G	T	A	A
12048	A	C	A	T	T	T	G	A
11142	A	C	A	T	A	T	G	A
11091	A	C	A	T/G	G/A	T	G	A
11085	A	C	A	A/G	G	T	A	A
11083	A	C	A	T	G	T	G	A
11077	A	C	A	C	G	T	G	A
11073	A	C	A	A	T	T	A	A
11072	A	C	A	A	G	T	A	A
11071	A	C	A	T	T	T	G	A
11065	A	C	A	T	T	T	G	A
11057	A	C	A	T	T	T	G	A
11056	A	C	A	A	G	T	G	A
11050	A	C	A	T	G/A	T	A/G	A
11039	A	C	A/T	T	G	T	A	A
11022	A	C	T	T	A	T	G	A
2402	A	C	A	T	A	T	G	A

**Table 5:** Consistency rate of DST and mPCR-MS mini-sequencing technology.

Antibiotics	mPCR-MS Mini-Sequencing Technology Results	DST Results		Consistency Rate
		Resistance (Count)	Susceptibility (Count)	
AMX	mutation (count)	0	3	95.5%
	non-mutation (count)	0	63	
MOX	mutation (count)	51	0	77.3%
	non-mutation (count)	15	0	
LEV	mutation (count)	43	8	68.2%
	non-mutation (count)	13	2	
CLA	mutation (count)	37	0	93.9%
	non-mutation (count)	4	25	
AZI	mutation (count)	37	0	92.4%
	non-mutation (count)	5	24	
TET	mutation (count)	1	0	97.0%
	non-mutation (count)	2	63	

**Table 6:** Target genes with mutation sites of six antibiotics.

Antibiotics	Genes	Mutation Sites	Mutations
AMX	*pbp1A*	1240	A→C
		1667	C→G
		1684	A→T
LEV	*gyrA*	261	C→T/A/G
		271	G→A/T
MOX		573	T→G/C
CLA	23S *rRNA*	2143	A→G
AZI			
TET	16S *rRNA*	928	A→C

**Table 7:** Mass probes of variant target sites.

Genes	Mutation Sites	Mass Probes	Mass Probe Tubes
23S *rRNA*	2143	HP BP3	B ^a^-23S *rRNA*-2143F
*pbp1A*	1667	HP AP1a	A ^a^-*pbp1A*-1667Fa
		HP AP1b	A-*pbp1A*-1667Fb
	1684	HP AP5a	A-*pbp1A*-1684Ra
		HP AP5b	A-*pbp1A*-1684Rb
	1240	HP AP2a	A-*pbp1A*-1240Fa
		HP AP2b	A-*pbp1A*-1240Fb
*gyrA*	261	HP BP4	B-*gyrA*-261F
	271	HP AP4	A-*gyrA*-271F
	573	HP AP3	A-*gyrA*-573R
16S *rRNA*	928	HP BP6	B-16S *rRNA*-928R

^a^: A and B represent that the mass probe has divided into two tubes; tube A contained AP1-5 and tube B contained BP3, 4, 6.

**Table 8:** Primer sequences for variant sites of target genes.

Genes	Mutation Sites	Primer Name	Fixed Sequence	Primer Sequences (5′-3′)	Complete Primer Sequences (5′-3′)
23S *rRNA*	2143	HP 1F	acgttggatg	GGGAGCTGTCTCAACCAGAG	acgttggatgGGGAGCTGTCTCAACCAGAG
	2143	HP 1R	acgttggatg	CAAAGCCTCCCACCTATCCT	acgttggatgCAAAGCCTCCCACCTATCCT
*pbp1A*	1667 1684	HP 2F	acgttggatg	GGAGTTTGGCTCGCATTAAA	acgttggatgGGAGTTTGGCTCGCATTAAA
	1667 1684	HP 2R	acgttggatg	GCCAATAGGCGTGTTATCGT	acgttggatgGCCAATAGGCGTGTTATCGT
	1240	HP 3F	acgttggatg	GCGCGAAAYTTTGAAAATG	acgttggatgGCGCGAAAYTTTGAAAATG
	1240	HP 3R	acgttggatg	AGCCAAGCTGATCGCTTAAA	acgttggatgAGCCAAGCTGATCGCTTAAA
*gyrA*	261 271	HP 4F	acgttggatg	TAGGATCGTGGGTGATGTGA	acgttggatgTAGGATCGTGGGTGATGTGA
	261 271	HP 4R	acgttggatg	CAGCGTTATCGCCATCAATA	acgttggatgCAGCGTTATCGCCATCAATA
	573	HP 5F	acgttggatg	AGCCGTCTGCCTAACCTTTT	acgttggatgAGCCGTCTGCCTAACCTTTT
	573	HP 5R	acgttggatg	TCAGGCCCTTTGACAAATTC	acgttggatgTCAGGCCCTTTGACAAATTC
16S *rRNA*	928	HP 6F	acgttggatg	CGGTCGCAAGATTAAAACTCA	acgttggatgCGGTCGCAAGATTAAAACTCA
	928	HP 6R	acgttggatg	GCAGCACCTGTTTTCAAGGT	acgttggatgGCAGCACCTGTTTTCAAGGT

## Data Availability

Data is contained within the article and Supplementary Materials.

## Supplementary Materials

The following supporting information can be downloaded at: https://www.mdpi.com/article/10.3390/ijms26041632/s1.
