# Supplementary material for: Multiplex PCR–Mass Spectrometry Mini-Sequencing Technology Detected Antibiotic Resistance of Helicobacter pylori to Six Antibiotics
Source: Int J Mol Sci. 2025 Feb 14;26(4):1632. doi: 10.3390/ijms26041632 (PMC11855914; doi:10.3390/ijms26041632)
Supplement: Supplementary file 1 [file ijms-26-01632-s001.zip › ijms-3409279-supplementary.pdf]

Supplementary

Figure S1. Sanger sequencing results at four sites of eight strains with bimodal peaks.

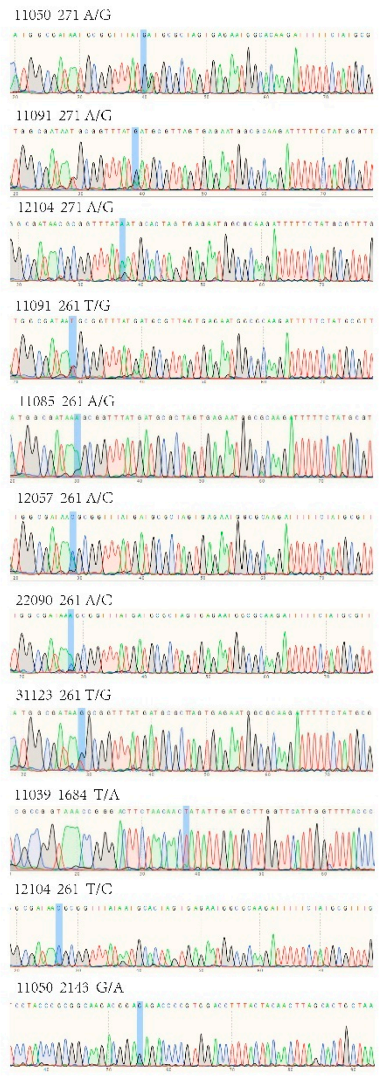

Table S1. DST and mPCR-MS mini-sequencing technology results of MET.

Strains	DST		Mutation Site
	MIC (≥8)	R/S	*rdxA* C148T
32141	＞256	R	C
32130	＞256	R	C
32126	0.5	S	C
32115	＞256	R	C
32078	2	S	C
32041	0.38	S	C
31135	＞256	R	C

31123	＞256	R	C
31078	＞256	R	C
31073	＞256	R	T
31058	＞256	R	C
22105	＞256	R	C
22104	＞256	R	C
22103	＞256	R	T
22100	＞256	R	C
22098	96	R	C
22096	0.75	S	C
22092	＞256	R	C
22090	0.75	S	C
22089	＞256	R	C
22075	＞256	R	T
22070	＞256	R	C
22061	＞256	R	C
22059	＞256	R	C
22058	0.75	S	C
22051	0.5	S	C
22049	1	S	C
22027	＞256	R	C
22024	＞256	R	C
21076	＞256	R	C
21072	＞256	R	C
21062	3	S	C
21018	＞256	R	C
12142	＞256	R	C
12137	＞256	R	C
12133	＞256	R	C
12132	＞256	R	C
12130	＞256	R	C
12128	＞256	R	C
12122	6	S	C
12118	＞256	R	C
12114	3	S	C
12110	＞256	R	C

12104	＞256	R	C
12094	＞256	R	C
12086	＞256	R	C
12073	1.5	S	C
12058	＞256	R	C
12057	0.38	S	C
12050	96	R	C
12048	＞256	R	C
11142	＞256	R	C
11091	12	R	C
11085	0.5	S	C
11083	48	R	C
11077	＞256	R	C
11073	＞256	R	C
11072	＞256	R	C
11071	＞256	R	C
11065	＞256	R	C
11057	＞256	R	C
11056	64	R	C
11050	＞256	R	C
11039	＞256	R	C
11022	＞256	R	C
2402	48	R	C

Table S2. Primers and MPE extension bases of mutation site of rdxA for MET resistance.

Mutation	Forward Primer	Reward Primer	MPE (5' – 3')	Mass Probe Mass (Da)	Extension Call	Mass Probe Mass (Da)	Extension Call	Mass Probe Mass (Da)
148 C/T	acgttggatg CGCTGAA ATCGCYA GRCTAT	acgttggatg ACGCTGA AGCGCTT TTAATC	CCAAGCTCTTA CAACACG	5412.6	C	5685.6	T	5754.6
